# Quality of life in Saudi Arabia: a patient-reported outcome measurement information system (PROMIS) – 10 global health study

**DOI:** 10.1186/s12889-025-22674-8

**Published:** 2025-05-06

**Authors:** Rami Al-Jafar, Razan AlGhassab, Haya M. Alzeer, Abdullah Al-Zeer, Mohammed S. Aldossary, Weam M. Banjar, Esraa Alnazzawi, Tala Althenayan, Dana Alotaibi, Abdulaziz Aljandal, Ahmed Alkhalifah, Shahad M. Alhajri, Malak Almasoud, Meshari Alnuwaiser, Abdulrahman Aljumah, Deemah Alabdulaali, Fahad Alsaawi, Khalid Alrajhi

**Affiliations:** 1 Data Services Sector, Lean for Business Services, 8th floor, 3rd Tower, Digital city, Riyadh, Saudi Arabia; 2 School of Public Health, Imperial College London, London, UK; 3 General Directorate of Research and Studies, Ministry of Health, Riyadh, Saudi Arabia; 4 Global Health Indicators Unit And Reports, Ministry of Health, Riyadh, Saudi Arabia; 5 King Khalid University Hospital, Riyadh, Saudi Arabia; 6 University of Galway, Galway, Ireland; 7 Taibah University, Medina, Saudi Arabia; 8 King Faisal Specialist Hospital and Research Center, Riyadh, Saudi Arabia

**Keywords:** Quality of life, Health-related quality of life, Self-reported, PROMIS, Saudi Arabia, Patient-reported outcome measures

## Abstract

**Background:**

Measuring the quality of life of a population provides a baseline for future comparisons and is essential for decision-makers, especially regarding resource allocation. Despite substantial investment in healthcare services in Saudi Arabia, no study has captured the general Saudi population’s overall health-related quality of life. This study utilized existing healthcare system data to develop reference values for the Patient-Reported Outcome Measurement Information System - Global Health (PROMIS-GH) survey for the population in Saudi Arabia and to examine associated sociodemographic predictors of health-related quality of life.

**Methods:**

From a nationwide survey conducted by the Saudi Ministry of Health, records of 40,000 out of 37,160,000 individuals were randomly selected with stratification for sex, age groups and regions. Participants received notifications via the national digital health platform (Sehhaty) to complete the PROMIS-GH survey.

**Results:**

A total of 3,630 individuals filled out the survey (response rate of 9.1%); the mean age was 38.6 ± 12.1 years, and 60.1% (*n* = 2182) were men. The general Saudi population had mean T-scores of 50.5 (± 10.3) for global mental health and 48.5 (± 9.8) for global physical health, both classified as “very good” based on the established Saudi-specific thresholds. Being Saudi or female was associated with lower physical and mental health scores.

**Conclusion:**

The health-related quality of life scores in Saudi Arabia are classified as “very good”; however, disparities exist. Future studies are needed to further investigate the reasons behind the sociodemographic and regional variations in HRQoL among the general population of Saudi Arabia.

## Introduction

The Kingdom of Saudi Arabia is undergoing economic and social changes that are impacting population health outcomes through the Health Sector Transformation Program under Vision 2030, which is reshaping preventive care services, digital health solutions, healthcare accessibility, and private sector participation in healthcare delivery [1]. The World Health Organization defines quality of life (QoL) as individuals’ perception of their position in life within their cultural context and value systems, relating to their goals and expectations [2]. Health-related QoL (HRQoL) reflects how the health status of a person affects their QoL [3, 4]. The prevalence of disease in the Kingdom is regularly studied and monitored; however, the impact of this burden on HRQoL of individuals remains elusive [5–7]. The absence of comprehensive HRQoL studies in the general Saudi population represents a critical knowledge gap, which is particularly crucial now as Saudi Arabia is continuously implementing major healthcare reforms under Vision 2030 [1]. Understanding population-level HRQoL can offer insights regarding the effectiveness of these reforms and identify areas requiring intervention [8]. The assessment of HRQoL could be done using multiple validated instruments, including the Patient-Reported Outcome Measurement Information System (PROMIS) Global Health (GH) [9]. PROMIS-GH, also referred to as PROMIS-10, is included in the standard set for Overall Adult Health (OAH) by the International Consortium for Health Outcomes Measurement (ICHOM). OAH encompasses health outcomes that are relevant to all adults, whether healthy or with controlled/uncontrolled diseases [10]. The PROMIS-GH survey contains 10 items that measure global physical health (GPH) and global mental health (GMH) T-scores. While PROMIS-29 provides a more comprehensive assessment of HRQoL through additional domains, PROMIS-GH is more feasible for large-scale population surveys because it is shorter, which aids in reducing respondent burden and increasing completion rates of the survey [11, 12].

PROMIS-GH has been used in various populations, ranging from specific demographic groups such as pregnant and postpartum women [13] and older adults [14] to people with specific health issues, including patients with stroke [15], musculoskeletal and orthopedic conditions [16–18], amyloidosis [19], autoimmune disease [20], and inflammatory bowel disease [21]. Additionally, it was used in general population studies of several countries, such as the United States, Netherlands, and Hungary [22–24]. Although other PROMIS scales have been used in Saudi Arabia, for example, PROMIS-29 has been validated in rheumatic disease patients, and the PROMIS General Life Satisfaction Short Form has been validated in the general population [25, 26]. To the best of our knowledge, no publications have documented PROMIS-GH use in Saudi Arabia to date. Using existing healthcare system data in Saudi Arabia, this retrospective analysis aimed to establish GMH and GPH T-score reference values and interpretability thresholds for the general population of Saudi Arabia and examine demographic and regional variations in HRQoL.

## Materials and methods

### Study design

The Saudi Ministry of Health (MoH) conducted a nationwide survey to inform decision makers during the year 2022, recognizing the research potential of this data, the authors obtained ethical approval (Date 30-07-2023/No: 23–60 E) to conduct a retrospective analysis study. The authors accessed the data retrospectively in August 2023, with all personal identifiers removed to ensure confidentiality. All research procedures adhered to the Declaration of Helsinki principles.

### Assessment tool

The PROMIS-GH survey comprises 10 categorical rating scale questions that measure overall GPH and GMH scores. PROMIS-GH yields standardized T-scores with a mean of 50 and a standard deviation (SD) of 10 for each of the two GMH and GPH dimensions. The PROMIS T-scores have a reference score of 50, which is the mean score for the United States (US) general population [27]. The GPH and GMH T-scores typically range from 20 to 80, with separate domain-specific cut-off points used to classify T-scores into five categories, which are poor, fair, good, very good, and excellent [28, 29].

### Study population and sampling

The records of 40,000 out of 37,160,000 individuals were selected randomly with stratification for sex, age groups, and regions from two sources: the MoH’s established registry and the database of Sehhaty platform (a national digital health platform serving all individuals in Saudi Arabia). The inclusion criteria were being 18 years or older and having at least one documented healthcare encounter in the national healthcare system.

### Data collection and scoring

The selected individuals received a notification via the Sehhaty platform app with a link directing them to an online survey containing Arabic and English versions of the PROMIS-GH (version 1.2) survey [30]. No subsequent follow-up/reminder notifications were sent. After data collection, data were shared with the Health Measures Scoring Service [10] to obtain summed raw scores and T-scores for GMH and GPH for each respondent.

### Statistical analysis

The GMH and GPH T-scores are presented as mean ± SD with a 95% confidence interval (CI). We examined differences in GPH and GMH T-scores using multiple linear regression models, with age group, sex, nationality, and health cluster region as independent variables. The models’ results were presented as mean differences with 95% CI. We established Saudi-specific interpretability thresholds for GPH and GMH T-scores following the methodology detailed in the Dutch population reference study, which elaborated on the approach originally used for the US reference population [24]. This involved categorizing participants into five groups based on their responses to the first question in the PROMIS-GH survey (“In general, would you say your health is excellent, very good, good, fair or poor”), calculating mean T-scores for each group, and then setting the thresholds at the midpoint between adjacent means. A *p*-value of less than 0.05 was considered statistically significant. Statistical analyses were conducted using R (version 4.3.0).

## Results

**Fig. 1:**
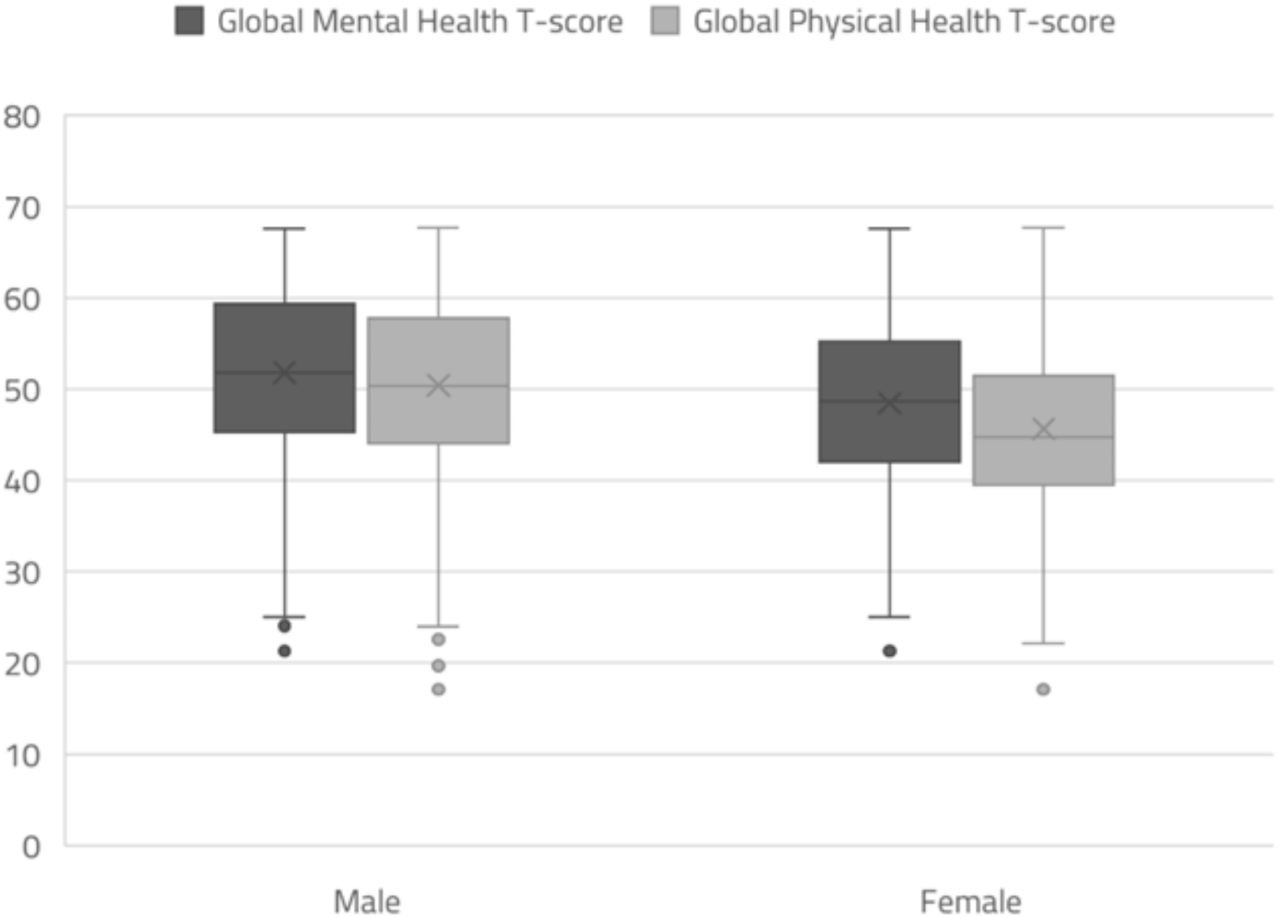
Variability of PROMIS GMH and GPH T-scores by sex

**Fig. 2:**
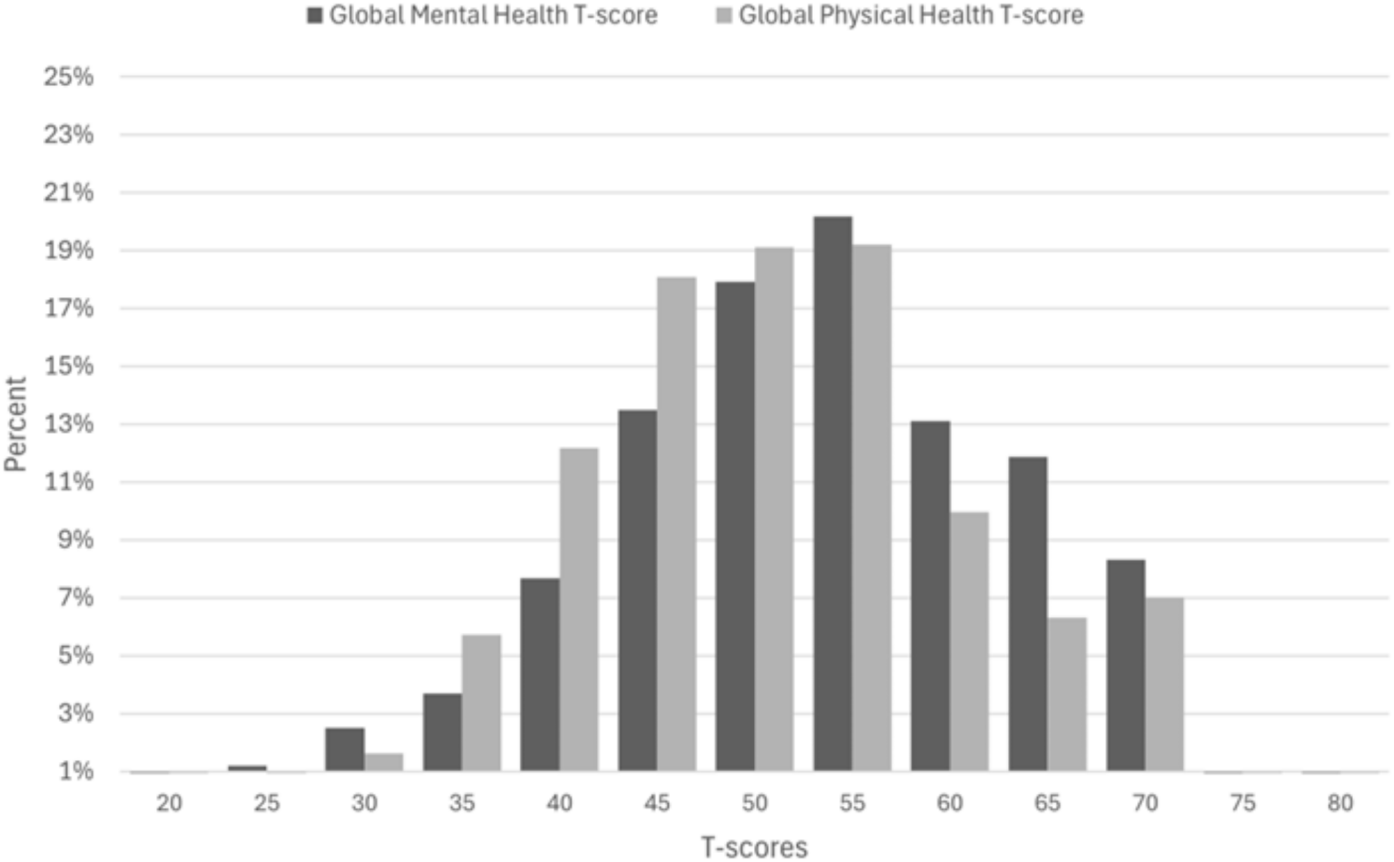
Distribution of PROMIS GMH and GPH T-scores

A total of 3,630 individuals completed the survey. The mean age was 38.6 ± 12.1 years, 2,182 (60.1%) of respondents were men, and the response rate was 9.1%. Table 1 presents the characteristics of the participants. The mean (SD) GMH and GPH T-scores were 50.5 (± 10.3) and 48.5 (± 9.8), respectively. The GPH T-score was lower in older age groups; individuals aged 70–97 years had the lowest GPH T-score (43.3), while the highest score (49.02) was observed amongst the 30–40 age group. The highest GMH T-score was 52.3 in the 50–60 years age group, and the lowest score was 48.2 in individuals aged 70 years and over (Table 1). Both GMH and GPH T-were higher in males at 51.82 (± 10.26) and 50.42 (± 9.73) compared to females at 48.46 (± 9.99) and 45.66 (± 9.26), respectively (Fig. 1). The distributions of GMH and GPH T-scores are presented in Fig. 2.

**Table 1:** Participant demographics and PROMIS GMH and GPH T-scores

Group (*n*)	Age (Mean ± SD)	Male (%)	Global Mental Health (GMH) T-score (Mean ± SD)	Global Physical Health (GPH) T-score (Mean ± SD)
All (3630)	38.61 12.06	60.1%	50.48 ± 10.28	48.52 ± 9.83
**Sex**				
Male (2182) Female (1448)	39.50 ± 12.11 37.27 ± 11.85	NA NA	51.82 ± 10.26 48.46 ± 9.99	50.42 ± 9.73 45.66 ± 9.26
**Age group**				
19–30 yrs (1015) 30–40 yrs (1202) 40–50 yrs (775) 50–60 yrs (440) 60–70 yrs (164) 70–97 yrs (34)	25.11 ± 3.04 35.53 ± 2.82 44.91 ± 2.89 54.74 ± 2.78 64.01 ± 2.18 75.71 ± 6.07	52% 63% 62% 64% 67% 79%	49.18 ± 10.97 50.44 ± 10.44 51.05 ± 10.01 52.39 ± 8.87 51.42 ± 8.36 48.22 ± 10.09	48.78 ± 9.94 49.02 ± 10.0.7 48.47 ± 10.04 47.98 ± 8.93 45.99 ± 7.92 43.38 ± 8.74
**Nationality**				
Saudi (2375) Yemen (249) Egypt (201) Philippines (130) India (118) Pakistan (102) Other (455)	38 ± 12.66 37.37 ± 10.33 39.32 ± 10.34 42.40 ± 9.00 40.95 ± 10.18 39.80 ± 10.73 40.04 ± 11.49	50.9% 82% 84% 64% 93% 92% 69%	49.19 ± 10.49 52.94 ± 9.40 50.38 ± 9.27 56.11 ± 7.72 55.31 ± 9.30 56.48 ± 8.16 51.67 ± 9.65	46.89 ± 9.68 51.37 ± 9.36 49.49 ± 9.31 54.78 ± 7.97 53.65 ± 9.67 54.06 ± 8.14 50.64 ± 9.48
**Health Cluster Region**				
Central (1312) Western (1266) Eastern (633) Southern (248) Northern (171)	38.82 ± 12.34 38.93 ± 12.17 39.07 ± 11.64 35.71 ± 10.64 37.19 ± 11.91	57% 61% 61% 68% 59%	50.26 ± 10.57 50.38 ± 9.94 50.55 ± 10.00 51.62 ± 10.58 50.96 ± 11.21	48.57 ± 9.89 47.91 ± 9.69 48.73 ± 9.50 50.24 ± 10.35 49.39 ± 10.30

Moreover, both GMH and GPH T-scores were lower in women compared with men by 2.41 (95% CI =-3.11 to -1.72, *p*-value < 0.001) and 3.97 (95% CI = -4.62 to -3.33, *p*-value < 0.001), respectively. Further, the scores of Saudis were significantly lower than those of all other nationalities except Egyptians. Individuals from southern health clusters had higher GMH (1.84, 95% CI = 0.47 to 3.21, *p*-value < 0.001) and GPH T-scores (1.74, 95% CI = 0.47 to 3.02, *p*-value < 0.001) compared with those from central health clusters. No differences were observed in the health clusters of other regions (Table 2).

**Table 2:** Differences between adjusted means PROMIS GMH and GPH T-scores

	*N* (%)	Global Mental Health (GMH) T-score		Global Physical Health (GPH) T-score	
		Difference between adjusted means (95% CI)	*p*. Value	Difference between adjusted means (95% CI)	*p*. Value
**Age groups**					
19–30 yrs 30–40 yrs 40–50 yrs 50–60 yrs 60–70 yrs 70–97 yrs	1015 (28%) 1202 (33.1%) 775 (21.4%) 440 (12.1%) 164 (4.5%) 34 (0.9%)	(Reference) 0.47 − 0.37 to 1.31) 1.07 (0.13 to 2.01) 2.48 (1.35 to 3.61) 1.88 (0.24 to 3.54) -1.28 (-4.70 to 2.13)	0.277 0.025 < 0.001 0.025 0.461	(Reference) -0.85 (-1.63 to -0.06) -1.38 (-2.26 to -050) -1.83 (-2.88 to -0.78) -3.35 (-4.89 to -1.82) -6.23 (-9.41 to -3.04)	< 0.001 < 0.001 < 0.001 < 0.001 < 0.001
**Sex**					
Male Female	2182 (60.1%) 1448 (39.9%)	(Reference) -2.42 (-3.11 to -1.72)	< 0.001	(Reference) -3.97 (-4.62 to -3.33)	< 0.001
**Nationality**					
Saudi Egypt Yemen Philippines India Pakistan Other	2375 (65.4%) 201 (5.5%) 249 (6.9%) 130 (3.6%) 118 (3.3%) 102 (2.8%) 455 (12.5%)	(Reference) 0.41 (-1.06 to 1.87) 3.10 (1.77 to 4.43) 6.42 (4.64 to 8.21) 5.04 (3.17 to 6.91) 6.26 (4.26 to 8.27) 2.08 (1.06 to 3.09)	0.587 < 0.001 < 0.001 < 0.001 < 0.001 < 0.001	(Reference) 1.45 (0.09 to 2.81) 3.33 (2.09 to 4.57) 7.63 (5.97 to 9.29) 5.32 (3.57 to 7.07) 5.80 (3.94 to 7.67) 3.25 (2.30 to 4.19)	0.037 < 0.001 < 0.001 < 0.001 < 0.001 < 0.001
**Health Cluster Region**					
Central Western Eastern Southern Northern	1312 (36.1%) 1266 (37.8%) 633 (17.4%) 248 (6.8%) 171 (4.7%)	(Reference) 0.10 (-0.67 to 0.88) 0.07 (-0.87 to 1.03) 1.84 (0.47 to 3.21) 1.21 (-0.38 to 2.81)	0.788 0.869 0.008 0.136	-0.62 (-1.34 to 0.10) 0.01 (-0.88 to 0.89) 1.74 (0.47 to 3.02) 1.23 (-0.25 to 2.72)	0.093 0.984 0.007 0.103

**Fig. 3:**
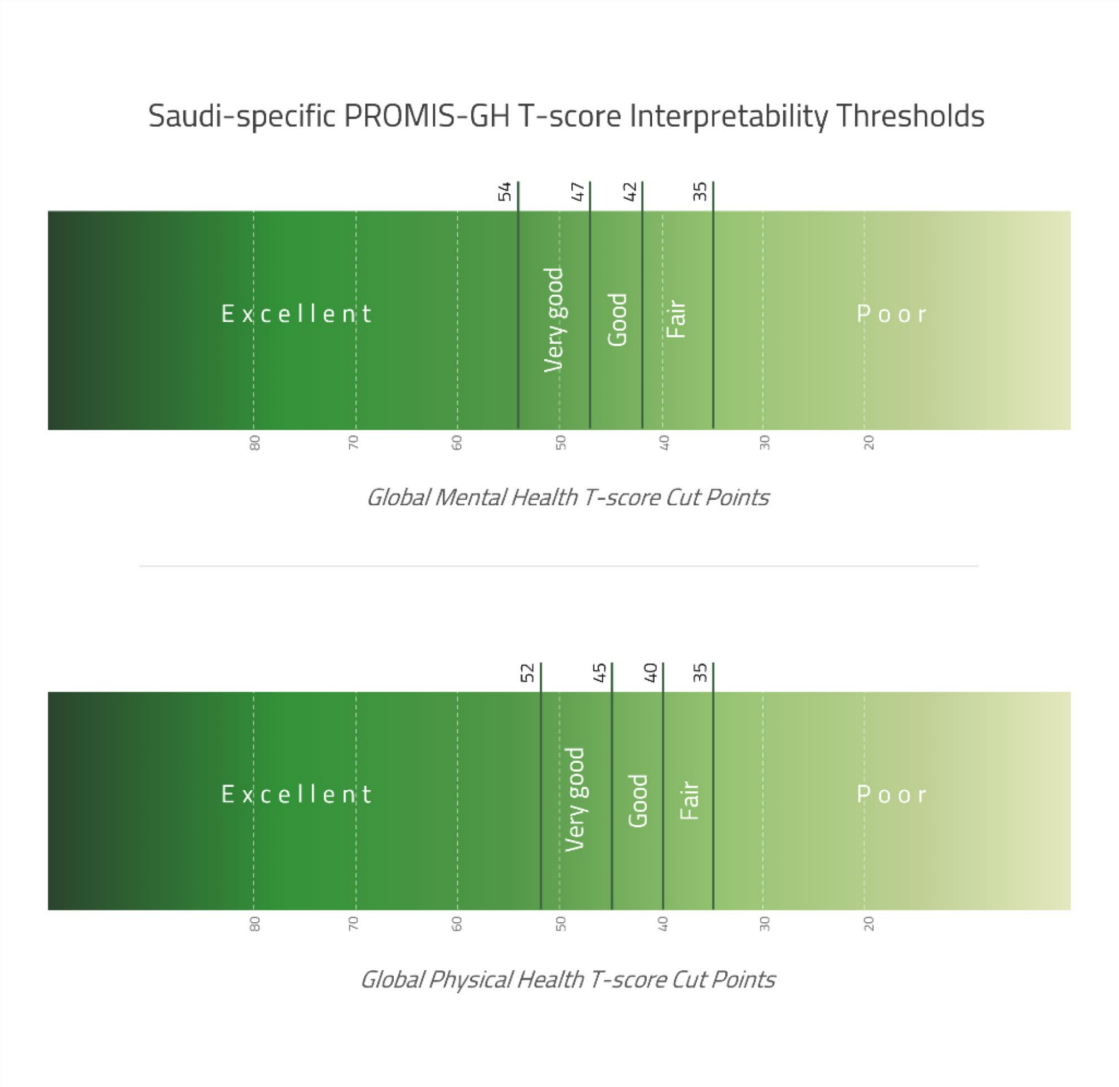
Saudi-specific PROMIS GMH and GPH T-scores interpretability thresholds

Additionally, we established Saudi-specific GMH and GPH T-scores interpretability thresholds, which classify T-scores into the following categories: poor, fair, good, very good, and excellent (Fig. 3).

## Discussion

Analysis of PROMIS-GH T-scores in the Saudi population revealed a mean GMH T-score of 50.48 and a mean GPH T-score of 48.52, both comparable to the US reference population T-score of 50. The GMH T-score is classified as “very good” according to both Saudi-specific and US reference thresholds, while the GPH T-score is classified as “very good” using Saudi-specific thresholds but as “good” using US reference thresholds [28]. Being male was associated with higher GPH and GMH T-scores. Age showed an inverse association with GPH T-scores. In contrast, GMH T-scores exhibited a positive association with age, reaching their peak in the 50–60 years age group before showing a gradual decline after 60 years. Moreover, being Saudi was associated with lower GPH and GMH T-scores compared with other nationalities living in Saudi Arabia. Individuals from the southern health cluster demonstrated better scores compared to the other health cluster regions in Saudi Arabia.

### Sociodemographic characteristics and HRQoL

Women were underrepresented in the sample compared to men, who comprised 60.1%, which may influence the interpretation and generalizability of our findings. A similar pattern was noticed in a large national study that targeted > 3.5 million individuals in Saudi Arabia using an online health-related survey through the Absher platform (Saudi Arabia’s official e-government platform), which reported a sample of 84% males [31]. In contrast to established literature demonstrating lower male participation rates in online surveys [32]. The disproportionate representation of women in our sample (40%) could have restricted our ability to fully capture these sex-based variations in HRQoL. Moreover, we found that both GMH and GPH T-scores were lower in women compared to men, in accordance with international studies that investigated PROMIS-GH T-scores in the Netherlands and Hungary [23, 24]. Similarly, previous studies using various HRQOL assessment tools in Saudi Arabia have shown that women consistently report lower scores than men and explained that this may be due to cultural constraints [33–35]. These constraints likely stem from a combination of sociocultural, economic, and healthcare-related factors [36]. Furthermore, obesity and overweight, which are more prevalent among Saudi women compared to Saudi men, are known contributors to lower HRQoL [37, 38].

Nationality is another important variable associated with HRQoL in Saudi Arabia. We found that the GPH T-score of Saudis was significantly lower than the scores of other nationalities. Similarly, Saudis’ GMH T-scores were lower than those of all other nationalities, except for Egyptians. This finding was expected since most non-Saudis are workers who have been medically examined in their home countries and tested again on arrival to gain a permit to work [39]. Additionally, most non-Saudis were men and more likely to belong to younger age groups to be eligible for work [40]. Thus, the non-Saudi groups are healthy groups not representative of their populations and any comparison between Saudis and non-Saudis in this paper should not be used to compare HRQoL across nationalities. Nevertheless, higher HRQoL scores among non-Saudis align with complex patterns documented internationally. In Germany, Turkish migrants demonstrated lower mental health scores relative to natives, while Polish migrants exhibited better physical health levels [41]. In Australia, migrants from English-speaking countries typically showed better outcomes than native Australians, while migrants from non-English speaking countries demonstrated poorer outcomes in relation to physical and mental health [42]. The cultural interpretations of health status may be attributed to these observed differences [43, 44].

The southern health clusters demonstrated significantly higher GMH and GPH T-scores compared to central health clusters. No significant differences were observed among health clusters in the northern, eastern, and western regions. Previous studies have reported mixed and inconsistent results regarding regional variations in factors that could affect mental and physical health outcomes in Saudi Arabia, with no consensus emerging from the literature [45–48]. This suggests that regional differences may be influenced by multiple complex factors that are yet to be defined locally. Assumptions regarding a decline in physical function with aging are predictable and were reflected accurately in low GPH T-scores in the 70–97 years age group. This finding could be a result of a variety of factors, including age-related declines in physical function and chronic health diseases [49, 50]. Older adults may also face barriers to healthcare services access, which affects their health outcomes [51].

### Country-level differences in HRQoL

The Saudi overall mean GPH and GMH T-scores (48.52 and 50.48) were comparable to the US reference population values (50.0 and 50.0) [28, 29]. Similarly, the Hungarian overall mean GPH T-score (49.0) was slightly lower than those of the US and Saudi reference values, and GMH T-score (47.7) was lower than those of the US and Saudi reference values [23]. The Dutch overall mean GPH and GMH T-scores (45.2 and 44.7) were lower than US and Saudi reference values [24]. Suggesting that both Saudi and Hungarian general populations demonstrated better HRQoL than the Dutch population. These differences should be interpreted with caution, as they may reflect variations in cultural perceptions of optimal health and/or thresholds for reporting health concerns and not HRQoL differences [43, 44].

The T-score interpretability thresholds established in the Saudi sample demonstrate notable variations from both Dutch and US reference thresholds. For GMH T-scores, the Saudi thresholds (Poor < 35 to Excellent ≥ 54) show higher minimum values than US thresholds (Poor < 29 to Excellent ≥ 56), but lower ones than Dutch standards (Poor < 38 to Excellent ≥ 56). All three populations share similar upper thresholds for the Excellent classification, suggesting agreement on higher-end GMH T-scores. As for GPH T-scores, Saudi thresholds (Poor < 35 to Excellent ≥ 52) maintain the same lower value as Dutch (Poor < 35 to Excellent ≥ 57) and US values (Poor < 35 to Excellent ≥ 58) but set a notably lower threshold for the Excellent classification [24, 28]. The different values observed across populations emphasize the importance of using population-specific thresholds to accurately interpret scores.

### Response rate

Although low response rates are predictable in online surveys, they could limit the generalizability of the results [52]. The low response rate (9.1%) could be attributed to several factors. First, the survey was conducted online through the Sehhaty platform, which may have limited accessibility for certain population sub-groups, particularly those facing digital literacy barriers or individuals with disabilities such as hearing or visual impairments. Second, some individuals may have been hesitant to participate due to concerns about privacy. Finally, the random selection process may have included individuals who were not interested in participating in the survey or who did not have the time or motivation to complete it. This may suggest selection bias in the sample. Since people with better HRQoL might have been too busy with their daily lives to participate. On the other hand, people who have health issues might have been more likely to complete the survey, possibly affecting the results. Higher response rates in follow-up studies are needed to confirm the findings. Notably, another national-level online health-related survey conducted on the Absher platform reported a low response rate of 24.6% [31].

### Strengths and limitations

This study has a number of strengths. To the best of our knowledge, this is the first study to use the PROMIS-GH survey on the general population of Saudi Arabia. Random stratified sampling was used in order to choose a representative sample. Moreover, the relatively large number of participants facilitated stratified analyses, such as comparisons of scores between Saudis and non-Saudis and across regions. As for the limitations, the response rate was low, and distributing the survey electronically could have excluded individuals lacking digital access, potentially contributing to sampling bias. While PROMIS-GH is a validated tool for measuring general health status through mental and physical health domains, it does not capture the broad range of health conditions that affect HRQoL. Additionally, there was a lack of sociodemographic data on factors that could have helped investigate the results further, and that may have been possible confounders, such as familial status, education level, income level, and rural/urban residence.

## Conclusions

HRQoL scores in Saudi Arabia are classified as “very good”; however, disparities exist. Being Saudi, female, and elderly were identified as the main risk factors for a lower HRQoL. The broader implications of these findings can inform national health policy development and resource allocation to address health disparities within Saudi Arabia. We recommend enhancing awareness of existing digital health solutions to improve healthcare accessibility and implementing targeted physical and mental health improvement interventions, particularly for vulnerable groups, to help address identified HRQoL disparities. While this study provides valuable insights, the findings should be interpreted with caution because the low response rate limits the findings’ generalizability. Future studies should employ strategies to achieve higher response rates to enhance generalizability and employ mixed-method approaches, including qualitative studies, to understand the reasons behind the sociodemographic and regional variations in HRQoL among the general population of Saudi Arabia.

## Data Availability

No datasets were generated or analysed during the current study.
